# Effect of cecal microbiota transplantation between different broiler breeds on the chick flora in the first week of life

**DOI:** 10.1016/j.psj.2021.101624

**Published:** 2021-11-28

**Authors:** Laura Glendinning, Cosmin Chintoan-Uta, Mark P. Stevens, Mick Watson

**Affiliations:** The Roslin Institute and Royal (Dick) School of Veterinary Studies, University of Edinburgh, Easter Bush, Midlothian, EH25 9RG, United Kingdom

**Keywords:** microbiota, broiler, caecum, transplant, 16S rRNA gene

## Abstract

The cecal microbiota plays numerous roles in chicken health and nutrition. Where such microbiota differs between lines exhibiting distinct phenotypes, microbiota transplantation offers scope to dissect the role of gut microbial communities in those traits. However, the composition and stability of transplants over time is relatively ill-defined and varying levels of success have been reported. In this study, we transplanted cecal contents from adult Roslin broilers into chicks from a different broiler line. Within <12 h posthatch, Ross 308 chicks received an oral gavage of cecal contents (n = 26) or a PBS control (n = 24). Cecal contents samples were collected postmortem from birds on d 1, 2, 3, 4, and 7 posthatch. DNA was extracted from these samples and the transplant inoculum and the V4 region of the 16S rRNA gene was amplified and sequenced. The cecal microbiota of chickens receiving the microbiota transplant was significantly different in composition and significantly richer and more diverse, in comparison to control birds. At the final timepoint (d 7), of the 150 Operational Taxonomic Units (**OTUs**) that were >0.1% abundant (average) in the donor sample, 137 were detected in the treated group (75 were >0.1% abundant (average)) while only 88 were detected in the control group (29 were >0.1% abundant (average)). Our data therefore suggests that stable transplantation of the cecal microbiota between lines is achievable using the methods described in this paper.

## INTRODUCTION

The ceca harbor the greatest concentration of microbes in the chicken gastrointestinal tract. These microbial communities are commonly referred to as the cecal microbiota. Despite advances in knowledge from metabarcoding, metagenomic and culturing studies, there are still many questions left to answer about the impact of the cecal microbiota on factors such as feed conversion efficiency and resistance to colonization by pathogens. Recent metagenomic studies have highlighted that there remain many uncultured and unstudied microorganisms in the chicken ceca (Glendinning et al., 2020; Gilroy et al., 2021; Segura-Wang et al., 2021), which may provide fruitful avenues for trait optimization through microbiota manipulation. One technique that can be used to assess the impact of the microbiota on key traits is microbial transplantation, where cecal contents or feces are taken from one bird and transplanted into another.

Studies have shown varying success when attempting to transplant gastrointestinal microbiota in chickens, potentially due to the variability of the methods used. Storage of cecal contents or feces prior to administration, or the exposure of these samples to oxygen, may impact the viability of some of the bacteria they contain. It would also be expected that the type of inoculum used will affect the likelihood of a successful transplantation.

The long-term impact of transplantation has also varied between studies. Metzler-Zebeli et al*.* demonstrated that administration of microbial transplants to young chicks induced long-term effects on their microbiota composition, though the recipient microbiota did not exactly reflect that of the donor (Metzler-Zebeli et al., 2019). However, a study by Ramírez et al. found that distinctions in microbiota compositions between chickens receiving microbial transplants and controls were present at d 7 posthatch, but were no longer present at d 14 (Ramírez et al., 2020). We have previously found that when transplanting cecal microbiota between 2 lines that were differentially resistant to *Campylobacter* infection, changes were observed in the microbiota composition (Chintoan-Uta et al., 2020). However, these changes were time dependent and at later timepoints the recipient bird line had a greater effect on the microbiota than the transplant.

In this study we sought to determine whether it was possible to transplant the cecal microbiota from adult 40-ek-old Roslin broilers to <12 h post hatch chicks from a different broiler line (Ross 308). We used freshly collected cecal contents as an inoculum, to provide as accurate a representation of the cecal microbiota as possible. Cecal contents were transplanted within 30 min of collection. We also administered the cecal inoculum within a few h of hatch to decrease the likelihood of chicks being exposed to environmental bacteria prior to transplantation. We then characterized the development of the cecal microbiota over the first 7 d of life relative to the donor inoculum and control birds, as this is the time period identified in previous studies as being most impacted by transplantation.

## MATERIALS AND METHODS

Animal procedures were performed in compliance with the Animals (Scientific Procedures) Act of 1986 and approved by the Animal Welfare and Ethical Review Board of The Roslin Institute. Donor birds weighing >3 kg were killed by captive bolt and recipient birds weighing <3 kg were killed by cervical dislocation in accordance with Schedule 1 of the Act. All chickens were hatched/housed at the National Avian Research Facility at The Roslin Institute, a Home Office accredited breeding establishment.

Contents were collected postmortem from the 2 ceca of three 40-we"?>k-old Roslin broilers (a closed inter-crossed population derived from a commercial Cobb female parent and the commercial Hubbard M99 male broiler). These were mixed and diluted in phosphate-buffered saline (PBS) at a 1:6 w:w dilution (Chintoan-Uta et al., 2020). Within 30 min of collection from donors, 100 µL of the cecal content suspension was administered to n = 26 Ross 308 chicks (<12 h posthatch) by oral gavage. A further 24 Ross 308 chicks received 100 µL of PBS, as a control group. Treated and control birds were split between 4 pens to control for pen effects: pen 1 contained 12 birds from the control group; pen 2 contained 13 birds from the control group; pen 3 contained 12 birds from the treatment group; and pen 4 contained 13 birds from the treatment group. Pens 1 and 2 were adjacent to one another, as were pens 3 and 4. Chickens were housed in the same room, in pens with wood shaving as bedding and received water and food (containing coccidiostats) ad libitum. A solid partition was placed between adjacent pens, which fully prevented transfer of fecal matter between pens. Floor drainage was in place between nonadjacent pens. New overshoes were used when entering each pen to prevent transfer of fecal matter.

Cecal contents were taken postmortem from chicks on d 1 [5 control chicks (pen 1: n = 2, pen 2: n = 3) and 5 treated chicks (pen 3: n = 2, pen 4: n = 3)]; d 2 [5 control chicks (pen 1: n = 3, pen 2: n = 2) and 5 treated chicks (pen 3: n = 3, pen 4: n = 2)]; d 3 [5 control chicks (pen 1: n = 2, pen 2: n = 3) and 5 treated chicks (pen 3: n = 2, pen 4: n = 3)]; d 4 [5 control chicks (pen 1: n = 3, pen 2: n = 2) and 5 treated chicks (pen 3: n = 3, pen 4: n = 2)]; and d 7 [4 control chicks (pen 1: n = 2, pen 2: n = 2) and 6 treated chicks (pen 3: n = 3, pen 4: n = 3)]. To collect cecal contents, the abdomens of the birds were sprayed with 70% ethanol and the ceca were exposed using sterile scissors. The ceca were removed using sterile scissors at the junctions between the ceca and the large intestine. Luminal contents from both ceca were manually expressed into the same tube, then mixed and stored at -20°C until DNA extraction.

DNA extraction reagent only controls (containing no sample) were produced for each DNA extraction batch, to act as negative controls (Pollock et al., 2018). A mock control (20 Strain Even Mix Whole Cell Material: ATCC MSA-2002, LGC Standards, UK) and duplicate samples of the mixed donor cecal contents were also included. DNA extraction, PCR amplification of the V4 region of the 16S rRNA gene (expected amplicon size ∼400 bp) and Illumina Miseq sequencing were performed as described previously (Glendinning et al., 2019) using 2 × 10 µL of cecal contents. Mothur (v.1.44.) was used for quality control, alignment, taxonomic assignment and clustering of Operational Taxonomic Unit (OTU) sequences, following an adjusted version of the Mothur Miseq pipeline (Kozich et al., 2013). Sequences were removed if they were <254bp, had ambiguous bases, did not align to the V4 region of the 16S rRNA gene, were not identified as bacteria, or contained homopolymers of >9 bp. Chimeras were identified using Vsearch (v.2.13.3.)(Rognes et al., 2016), then removed. The entire Silva database (v.138.)(Quast et al., 2012) was used for alignment (using the align.seqs command in Mothur) and taxonomic assignment. OTUs were clustered by similarity using the dist.seqs (cutoff = 0.03) and cluster commands from within Mothur (default parameters).

Statistical analyses were carried out in R (v.3.5.1.). Graphs were produced using the gpplot2 (v.3.2.1) and Venn Diagram (v.1.7.0) packages. The Vegan (v.2.5-6.) package was used to construct Nonmetric Multidimensional Scaling (**NMDS**) graphs using Bray-Curtis dissimilarity values. The Vegan package “adonis” function was used to perform PERMANOVA analyses. Richness (Inverse Simpsons) and diversity (Chao 1) indices were calculated and compared between groups using the Kruskal-Wallis test. ANCOM (Mandal et al., 2015) (v.2.1) was used to identify OTUs that were differentially abundant between groups, with the cut-off “detected_0.7.” Prior to statistical analyses samples were subsampled to 20,000 reads, except for ANCOM analyses.

The paired-read fastq files generated and analyzed during the study are available in the European Nucleotide Archive under project PRJEB46338. OTU tables can be found at https://doi.org/10.7488/ds/3129.

## RESULTS AND DISCUSSION

On average after quality control, 16S rRNA gene amplicons derived from samples of cecal contents from treated and control birds contained 167,259 ± 44,231 reads. Reagent only controls contained on average 673 ± 386 reads. Duplicate cecal donor samples contained 205,713 and 174,734 reads. Rarefaction curves for samples plateaued, indicating that the sequencing depth was adequate (Supplementary Figure 1). 10,416 OTUs were identified in our samples. All phyla present in the original mock community were present in the sequenced samples after subsampling (Supplementary Table 1), although members of the Actinobacteria were under-represented. All expected families were present, except for *Propionibacteriaceae* (*Cutibacterium acnes*). The most abundant chicken microbiota phyla (Bacteroidota and Firmicutes)(Rychlik, 2020) were well represented in our mock community.

**Figure 1:**
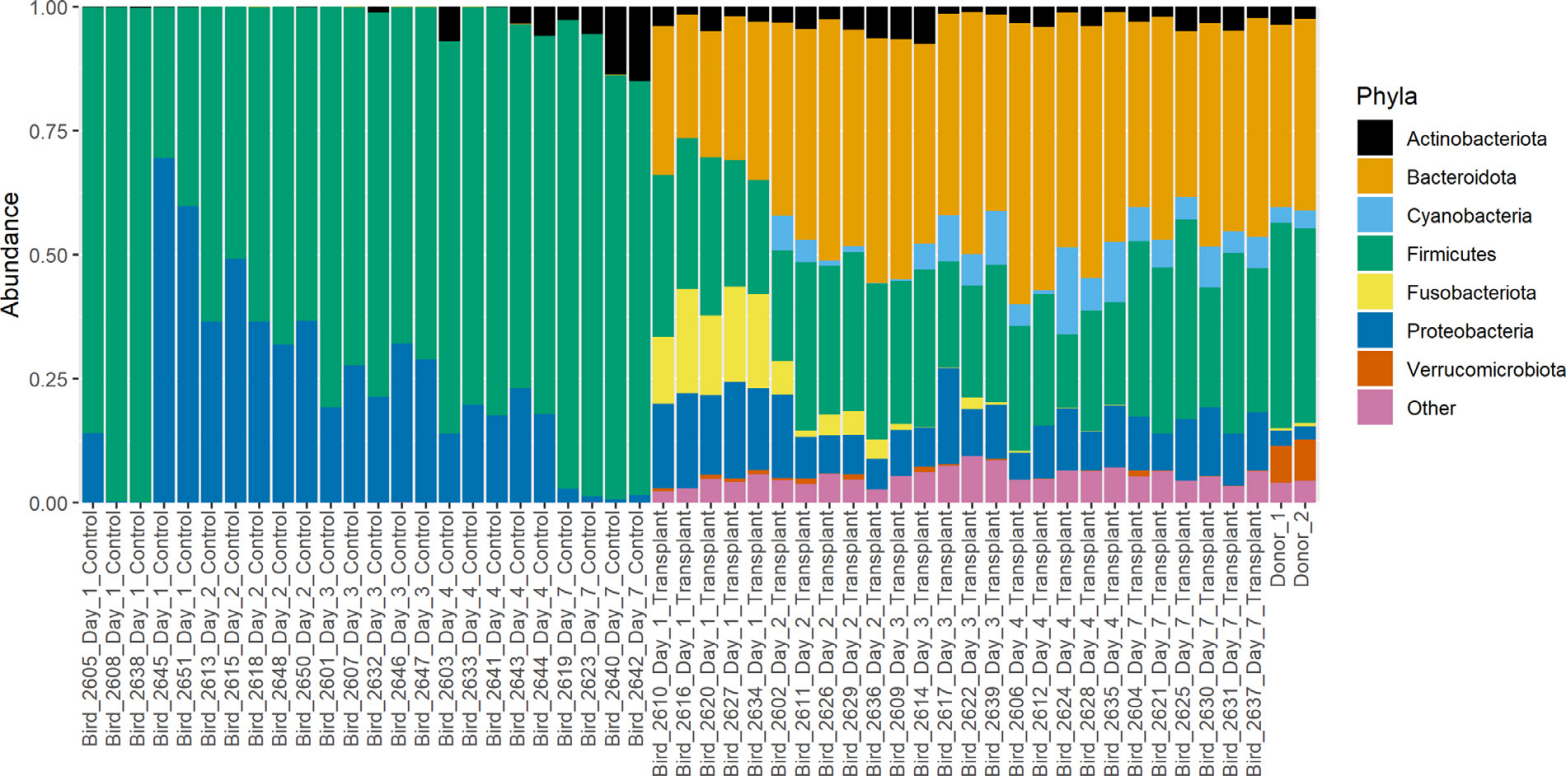
Barplot showing the proportion of bacterial phyla in each sample, after subsampling to 20,000 reads. Recipients of cecal transplants = Transplant. Control chickens that did not receive a cecal transplant = Control. Cecal transplant donors = Donor.

In the control group, 2 OTUs dominated the cecal microbiota at d 1: OTU3 (*Clostridium_sensu_stricto_1*: 41 ± 28%), and OTU1 (*Escherichia-Shigella*: 29 ± 34%) (Figure 1). At d 1 we have previously observed a similar *Escherichia* bloom in 2 different chicken lines (Chintoan-Uta et al., 2020) and a *Clostridium_sensu_stricto_1* bloom in Ross 308 birds (Glendinning et al., 2019). At d 2, the relative proportions of these OTUs decreased (OTU3: 5.3 ± 802%, OTU1:.20 ± 8.1%) while OTU2 (*Lachnospiraceae*_unclassified: 20 ± 11%) and OTU4 (*Enterobacteriaceae*_unclassified: 18 ± 12%) greatly increased in abundance. OTUs 1, 2 and 4 remained the most abundant OTUs at d 3 and 4. At d 7 the most abundant OTUs were OTU9 (*Bifidobacterium*: 8.3 ± 6.0%), OTU2 (*Lachnospiraceae*_unclassified: 5.4 ± 4.7%), OTU13 (*Lachnospiraceae*_unclassified: 5.4 ± 0.20%), OTU12 (*Lactobacillus*: 4.7 ± 1.7%), and OTU46 (*Ruminococcaceae*-Incertae_Sedis: 4.1 ± 0.62%). This progression over the first week, from a community dominated by *Clostridium_sensu_stricto_1* to a more diverse microbiota including members of the *Lachnospiraceae* and *Lactobacillaceae* reflects our previous findings in this chicken line (Glendinning et al., 2019).

**Figure 2:**
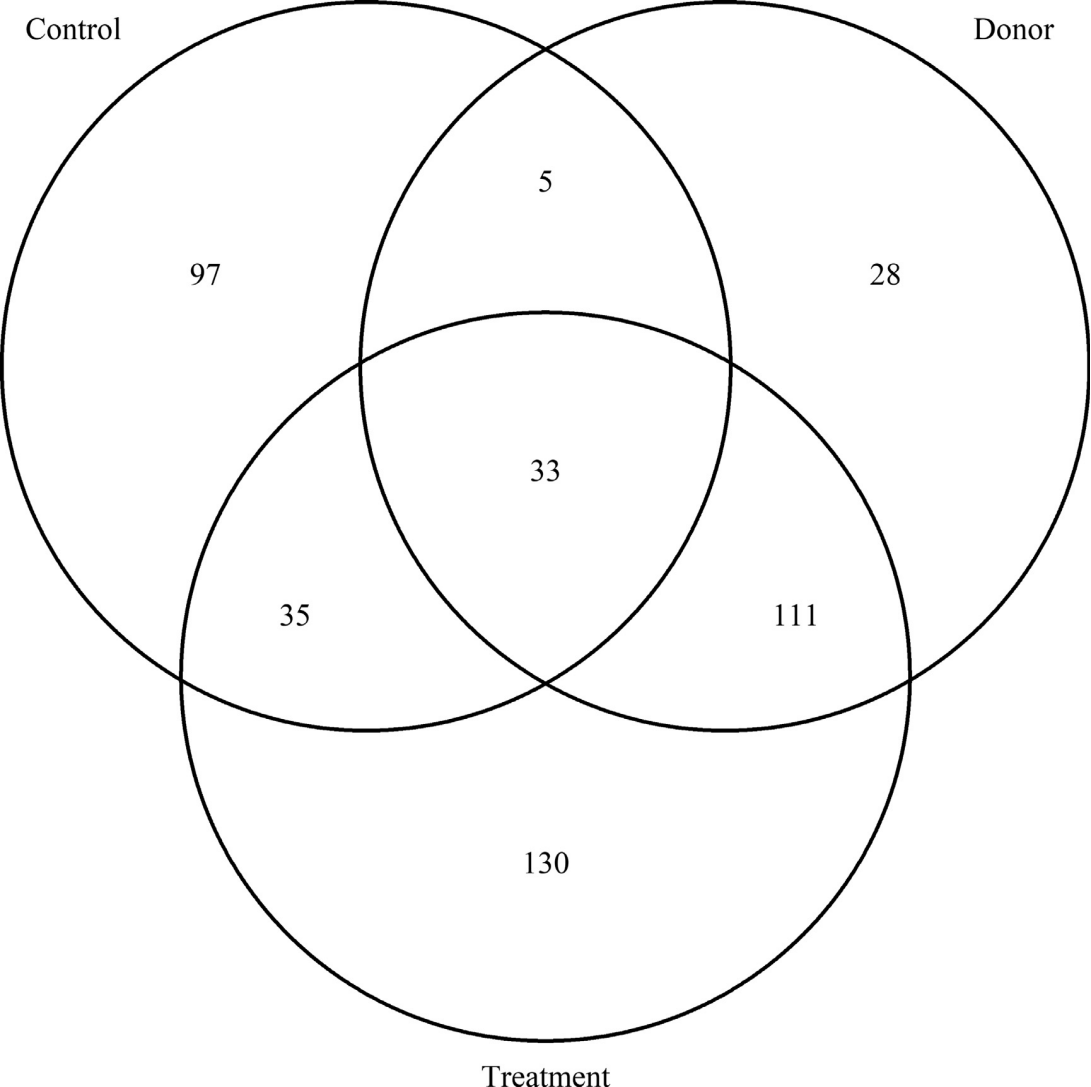
Venn diagram showing shared OTUs between cecal transplant donors (Donors: n = 2), recipients of cecal transplants (Treatment: n = 26) and control chickens that did not receive a cecal transplant (Control: n = 24). OTUs were counted as present in a group if they were at least 0.1% abundant (average) in at least one sample from the group, after subsampling to 20,000 reads per sample.

**Figure 3:**
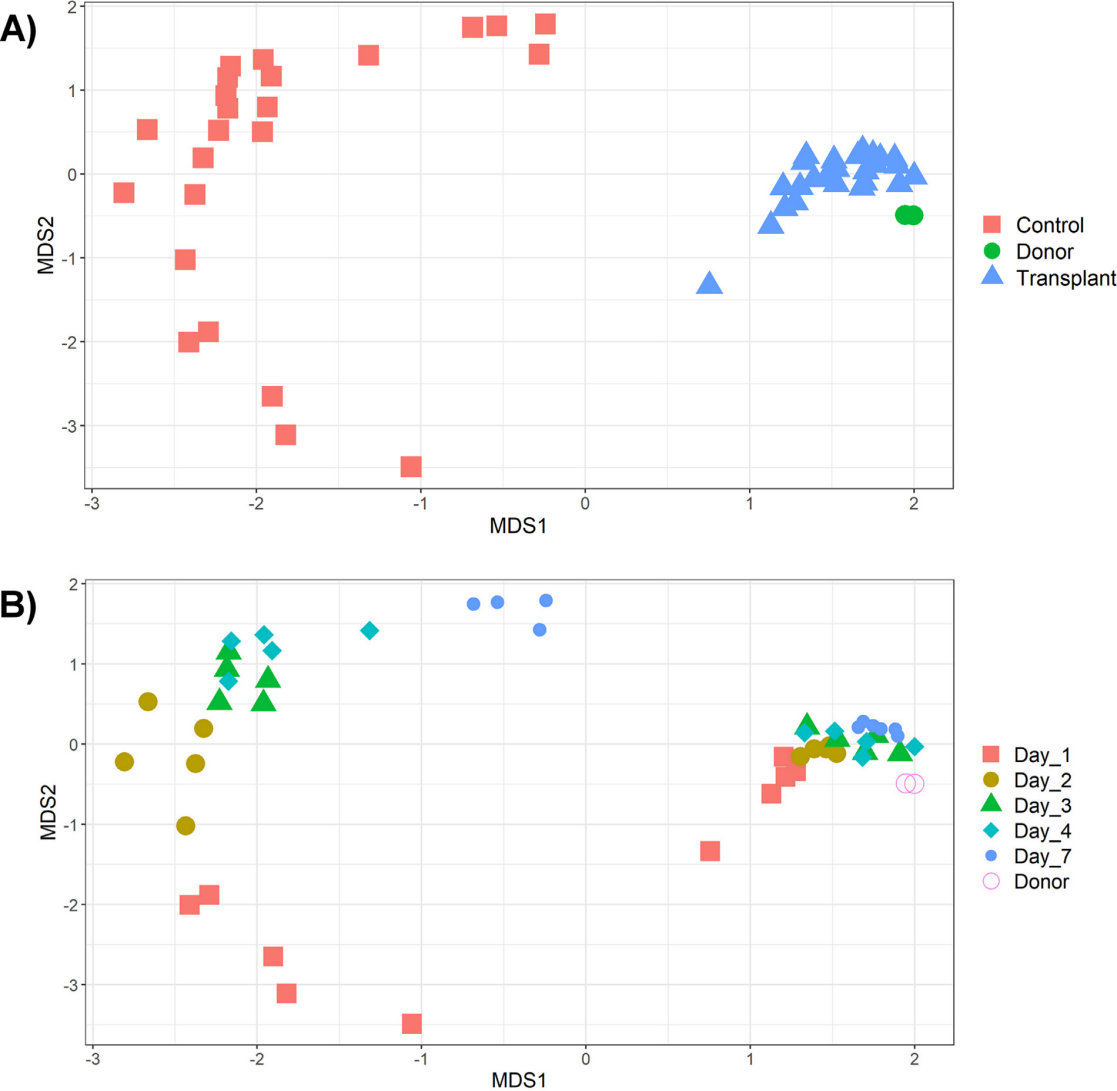
Bray–Curtis dissimilarity values were calculated and used to construct NMDS graphs showing sample clustering by microbiota composition. Stress = 0.07. (A) Samples labeled by treatment (AMOVA: *P* = 1e-05). Chickens received either PBS (Control) or a cecal transplant (Transplant) from donor birds (Donor). (B) Samples labeled by day of sampling posthatch (AMOVA: *P* = 1e-05).

In comparison, the cecal microbiota of birds that received microbial transplants was dominated by different bacterial OTUs. At d 1 the most abundant OTUs were OTU8 (*Fusobacterium*: 18 ± 3.1%), OTU1 (*Escherichia-Shigella*: 11 ± 2.2%), and OTU14 (*Bacteroides*: 7.1 ± 4.5%). Interestingly, these OTUs were far less abundant in the donor sample (OTU8: 0.54 ± 0.054, OTU1: 1.3 ± 0.16, OTU14: 0.82 ± 0.016). These OTUs may represent bacterial species from the donor or environment that is able to colonize the bird more quickly than other species. The most abundant OTUs at d 2 were OTU6 (*Bacteroidales*_unclassified: 6.0 ± 2.5%), OTU7 (*Phascolarctobacterium*: 6.0 ± 1.0%), OTU14 (*Bacteroides*: 4.3 ± 3.8%), OTU8 (*Fusobacterium*: 4.2 ± 2.0%) and OTU18 (*Bacteroidales*_unclassified: 3.5 ± 2.1%). Several of these OTUs were found in similar abundances in the donor sample: OTU6 (3.4 ± 0.14%), OTU7 (2.6 ± 0.4%) and OTU18 (2.3 ± 0.088%). At d 7, the most abundant OTUs were OTU10 (*Anaerobiospirillum*: 6.6 ± 1.8%), OTU16 (*Bacteroides*: 5.2 ± 1.6%), OTU7 (*Phascolarctobacterium*: 5.2 ± 1.1%), OTU15 (*Megamonas*: 5.0 ± 2.1%) and OTU6 (*Bacteroidales*_unclassified: 3.2 ± 0.32%). In the donor sample several of these OTUs were present in similar proportions while others were less abundant: OTU10 (0.084 ± 0.016%), OTU16 (0.72 ± 0.087%), OTU7 (2.6 ± 0.43%), OTU15 (0.15 ± 0.03%), and OTU6 (3.4 ± 0.14%). Overall, a greater abundance of OTUs were shared between the 2 donor samples and the treatment samples than between the donor samples and the control group (Figure 2). Several of the most abundant OTUs in the donor samples were only found in low abundances in treated samples. These included OTU42 (*Akkermansia*: 7.6 ± 0.47%), OTU128 (*UCG-005*: 3.0 ± 0.30%), OTU56 (*Rikenellaceae_RC9_gut_group*: 3.0 ± 0.080%), and OTU133 (*Paludibacteraceae*_unclassified: 2.7 ± 0.18%). Chicken cecal contents separated differently by their compositions according to whether they received the cecal transplant or the PBS control (*P* = 1e-05), with the treated samples clustering more closely to the donor samples (Figure 3). Samples also clustered significantly by time-point (*P* = 1e-05) (Figure 3). The interaction between treatment and time-point was also found to be significant (*P* = 1e-05). The donor sample contained on average 150 OTUs that were >0.1% abundant (average); at d 1, 136 of these OTUs were detected in the treated group [54 OTUs >0.1% abundant (average)] while 73 of these OTUs were detected in the control group [8 OTUs >0.1% abundant (average)]. At d 7, 137 of these OTUs were detected in the treated group [75 OTUs >0.1% abundant (average)] while 88 of these OTUs were detected in the control group [29 OTUs >0.1% abundant (average)]. This indicates that while some OTUs were not successfully transplanted, cecal content transplantation had a major impact on the microbiota, and on the development of the microbiota over time.

The most notable taxonomic difference between treated and untreated samples is the abundance of the phylum Bacteroidota, which is present at a high abundance in the donor sample (38 ± 1.4%). At d 1, this taxa was found at very low abundances in control samples (0.022 ± 0.0081%) but in high abundance in treated samples (28 ± 2.9%). By 7 d, the relative abundance of Bacteroidota in control samples had barely changed (0.020 ± 0.0039%), while in treated samples it had increased further (41 ± 4.4%). These results reflect those of Kubasova et al., who found that adult hens were efficient donors of Bacteroidetes when housed with young chicks (Kubasova et al., 2019).

**Figure 4:**
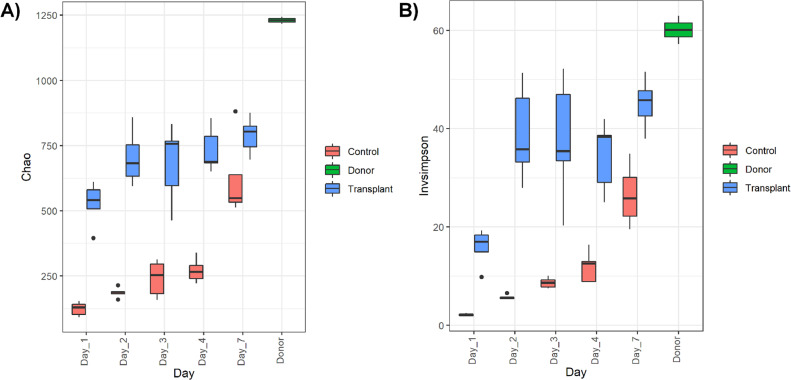
Richness and diversity of the cecal microbiota of chickens over the first 7 d of life. Chickens received either PBS (Control) or a cecal transplant (Transplant) from donor birds (Donor). (A) Richness of microbial communities, measured by Chao1 index. (B) Diversity of microbial communities, measured by the Inverse Simpsons Index.

At d 1, 114 OTUs were found to be differentially abundant between the treatment and control group. The number of OTUs differentiating the 2 groups then sharply increased at d 2 (226 OTUs), reflecting the increase in diversity of microbial communities over time (Figure 4). At d 3 and 4, 192 and 215 OTUs differentiated the groups respectively. The number of differentiating OTUs then sharply increased again at d 7 (364 OTUs).

The cecal microbiota of chickens that received a transplant of cecal contents had significantly higher richness (Kruskal-Wallis: *P* = 8.346e-08) and diversity (Kruskal-Wallis: *P* = 7.494e-08) than chickens that received control PBS. The diversity and richness of the microbiota increased over time for both groups, but did not reach the same levels as the donor communities by the end of the study period (Figure 4). This is to be expected, as the donor birds were 40-wk-old and therefore had a mature microbiota (Rychlik, 2020).

In conclusion, we were able to successfully transplant the majority of the bacterial members of the cecal microbiota from 40-wk-old broilers to chicks from a different broiler line. We observed significant differences in composition and diversity at all-time intervals sampled, and transplantation clearly changed the trajectory of cecal microbiota development. This technique could be used in future studies to transplant the microbiota between broiler lines with desired phenotypes, to assess the impact of the microbiota on those phenotypes.

## Appendix Supplementary materials

Image, application 1

Image, application 2

## References

[bib0001] Chintoan-Uta C., Wisedchanwet T., Glendinning L., Bremner A., Psifidi A., Vervelde L., Watson K., Watson M., Stevens M.P. (2020). Role of cecal microbiota in the differential resistance of inbred chicken lines to colonization by *Campylobacter jejuni*. AEM.

[bib0002] Gilroy R., Ravi A., Getino M., Pursley I., Horton D.L., Alikhan N.F., Baker D., Gharbi K., Hall N., Watson M., Adriaenssens E.M., Foster-Nyarko E., Jarju S., Secka A., Antonio M., Oren A., Chaudhuri R.R., La Ragione R., Hildebrand F., Pallen M.J. (2021). Extensive microbial diversity within the chicken gut microbiome revealed by metagenomics and culture. PeerJ.

[bib0003] Glendinning L., Stewart R.D., Pallen M.J., Watson K.A., Watson M. (2020). Assembly of hundreds of novel bacterial genomes from the chicken caecum. Genome. Biol..

[bib0004] Glendinning L., Watson K.A., Watson M. (2019). Development of the duodenal, ileal, jejunal and caecal microbiota in chickens. Anim. Microbiome..

[bib0005] Kozich J.J., Westcott S.L., Baxter N.T., Highlander S.K., Schloss P.D. (2013). Development of a dual-index sequencing strategy and curation pipeline for analyzing amplicon sequence data on the MiSeq Illumina sequencing platform. AEM.

[bib0006] Kubasova T., Kollarcikova M., Crhanova M., Karasova D., Cejkova D., Sebkova A., Matiasovicova J., Faldynova M., Pokorna A., Cizek A., Rychlik I. (2019). Contact with adult hen affects development of caecal microbiota in newly hatched chicks. PLoS One.

[bib0007] Mandal S., Van Treuren W., White R.A., Eggesbø M., Knight R., Peddada S.D. (2015). Analysis of composition of microbiomes: a novel method for studying microbial composition. Microb. Ecol. Health. Dis..

[bib0008] Metzler-Zebeli B.U., Siegerstetter S.-C., Magowan E., Lawlor P.G., O′Connell N.E., Zebeli Q. (2019). Fecal microbiota transplant from highly feed efficient donors affects cecal physiology and microbiota in low- and high-feed efficient chickens. Front Microbiol..

[bib0009] Pollock J., Glendinning L., Wisedchanwet T., Watson M. (2018). The madness of microbiome: attempting to find consensus “best practice” for 16S microbiome studies. Appl. Environ. Microbiol..

[bib0010] Quast C., Pruesse E., Yilmaz P., Gerken J., Schweer T., Yarza P., Peplies J., Glöckner F.O. (2012). The SILVA ribosomal RNA gene database project: improved data processing and web-based tools. Nucleic Acids Res.

[bib0011] Ramírez G.A., Richardson E., Clark J., Keshri J., Drechsler Y., Berrang M.E., Meinersmann R.J., Cox N.A., Oakley B.B. (2020). Broiler chickens and early life programming: microbiome transplant-induced cecal community dynamics and phenotypic effects. PLoS One.

[bib0012] Rognes T., Flouri T., Nichols B., Quince C., Mahé F. (2016). VSEARCH: a versatile open source tool for metagenomics. PeerJ.

[bib0013] Rychlik I. (2020). Composition and function of chicken gut microbiota. Animals.

[bib0014] Segura-Wang M., Grabner N., Koestelbauer A., Klose V., Ghanbari M. (2021). Genome-resolved metagenomics of the chicken gut microbiome. Front. Microbiol..

