# Supplementary figures and images for: Effect of cecal microbiota transplantation between different broiler breeds on the chick flora in the first week of life
Source: Poult Sci. 2021 Nov 28;101(2):101624. doi: 10.1016/j.psj.2021.101624 (PMC8704443; doi:10.1016/j.psj.2021.101624)

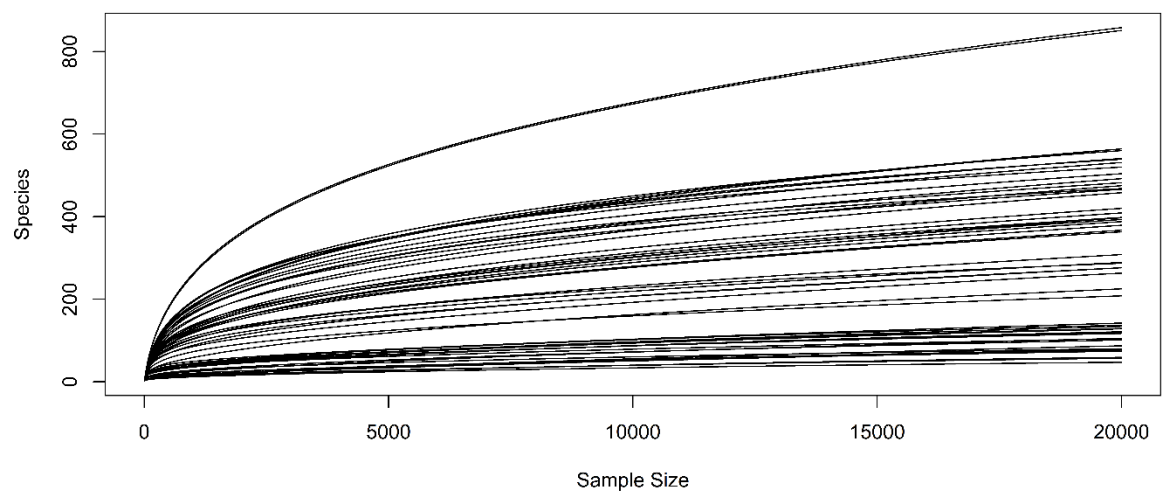

Supplementary figure 1: Rarefaction curves of all samples, sub-sampled at 20,000 reads.

Supplement: Supplementary file 1 [file mmc1.pdf]
